# Ethical Issues in Long-term Care in Low-, Middle- and High-Income Countries During the COVID-19 Pandemic

**DOI:** 10.1177/23337214221146660

**Published:** 2023-01-10

**Authors:** Preet Gandhi, Angel Petropanagos, Andreea Popescu, Darren Bugaresti, Theresa Nitti, Nipa Chauhan, Paula Chidwick, Jill Oliver

**Affiliations:** 1 University of British Columbia, Vancouver, Canada; 2 William Osler Health System, Brampton, ON, Canada

**Keywords:** long-term care, COVID-19, residents, ethical issues, visitation

## Abstract

Long-term care (LTC) centers experienced an unprecedented emergency involving exponential mortality during the COVID-19 pandemic. Individuals residing in long-term care were particularly vulnerable to the effects of COVID-19, placing residents, staff, families, and organizations in a precarious position. Complex issues surrounding how to manage vulnerable populations during the pandemic have highlighted the importance of gathering information on ethical issues that require effective policy and decision-making. This project sought to identify the ethical issues faced in long-term care by residents, families, staff, and organizations from stakeholders themselves. A total of 305 participants from 45 countries responded, highlighting numerous ethical issues in long-term care during COVID-19. While numerous issues were mentioned, there was an overlap in the themes of responses between stakeholders. Visitation, isolation, harm, staff well-being, and the overall enforcement of policies during the pandemic represented the most often discussed issues. As a preliminary study of this issue, future research is necessary in order to effectively guide pandemic policymaking moving forward.

## Introduction

The World Health Organization (WHO) declared the COVID-19 pandemic caused by the novel coronavirus SARS- COV2 on March 11, 2020. While at the time of writing, this pandemic continues to have a devastating impact on people around the world, the first and second waves of the pandemic were especially devastating for people living and working in long-term care (LTC) homes in Canada and around the world (Jolly, 2020; Oliver et al., 2020; Szczerbińska, 2020). In North America and Western Europe, literature clearly identifies that the majority of COVID-19 related deaths during those waves were in LTC homes (Faghanipour et al., 2020). The impacts on long-term care have continued over the course of the pandemic.

While several national and international reports have documented the multifactorial impact of COVID-19 on long-term care residents’ living conditions, to our knowledge, no literature currently offers *stakeholder-identified* ethical issues from across low, middle, and high income countries and different sociocultural contexts during the pandemic. As the pandemic developed, medical resource rationing created constraints on the ability of systems to respond, impacting the experiences of stakeholders in long-term care. Knowing the nature of the ethical issues identified by those working and living in LTC homes is key in informing the development of any ethical guidance. As such, an investigation into the ethical issues experienced in the global pandemic in LTC homes during public health emergencies was launched. Information about stakeholder experiences can help inform the development of effective and pragmatic policies, guidance, tools, and opportunities for collaboration that LTC homes and their various stakeholders might turn to during such emergencies.

Funded by the Pandemic and Emergency Preparedness Ethics Network with the World Health Organization, this ethics project sought to discover what self-identified stakeholders in LTC homes from across the globe felt were ethical issues caused by the pandemic, our team created a global online survey. This survey was distributed worldwide where we received responses from 45 countries, the bulk of them coming from Canada. The survey design included open-ended questions and intended to explore what was revealed by survey respondents in order to avoid any bias the developers might hold in understanding what would constitute an ethical issue. Through a content analysis, this study summarizes what stakeholders in LTC homes (including residents, staff, families, and organizations) identified as ethical issues.

## Methods

Using a participatory approach, a confidential and anonymous online four-question survey was created in English using SurveyMonkey. The survey was professionally translated into seven other languages (Arabic, Chinese, French, Hindi, Portuguese, Russian, and Spanish). Six languages were selected based on their designation of official languages of the United Nations, while Portuguese and Hindi were selected due to the number of individuals fluent globally (The United Nations, 2021). The survey was distributed globally and respondents were self-selected, as the survey was open to anyone who identified themselves as a stakeholder in LTC homes, anywhere in the world.

Through snowball recruitment, this survey was distributed via emails, online newsletters, social media platforms including Twitter, Facebook, and LinkedIn, as well as institutional announcements and a commentary published in online publications. To promote widespread distribution of surveys, we asked respondents to refer the survey to other persons believed to have an interest in responding and contributing (Kirchherr & Charles, 2018). Efforts were made to reach people in a variety of countries, particularly low- and middle-income countries in an attempt to ensure representation, including directly reaching out to federations which represent national associations, who in turn distributed surveys within their network (Kirchherr & Charles, 2018). This type of networking made it possible to reach global institutes, disseminating to over 20 countries. Invitations were sent in the various survey languages.

**Figure 1.**
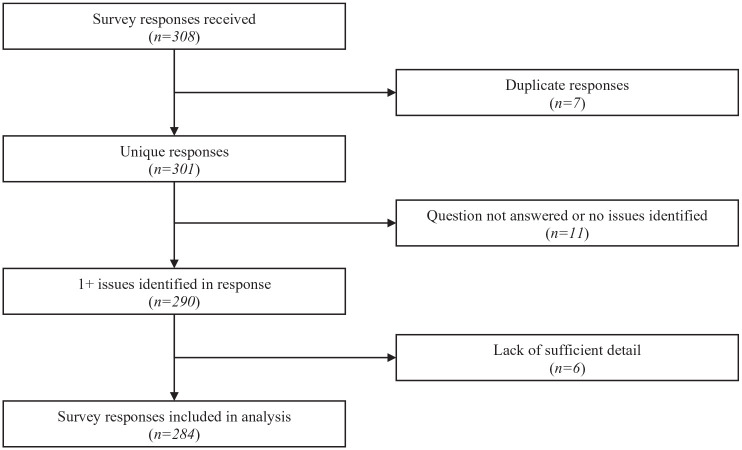
Survey response selection.

The survey was open between January 25th to March 1st, 2021. During this time, a total of 308 responses were recorded from 45 different countries (Figure 1). Non-English responses (the primary language of the reviewers) were flagged for translation using professional services to preserve the integrity of responses and minimize error. An initial review of the 308 responses identified 24 responses to be excluded due to duplication (*n* = 7), question not answered or no issues identified (*n* = 11), or lack of sufficient detail (*n* = 6). The remaining 284 responses were assumed to be from unique respondents and included in the analysis. Based on the call for projects that was solicited by the World Health Organization, wherein projects targeting global LTC stakeholders were sought, responses were then classified by country to identify LTC homes in high-income countries (HICs) and low-and middle-income countries (LMICs) using World Bank resources (The World Bank, 2021).

A content analysis on the remaining 284 responses identified themes and issues for further exploration. Content analysis was chosen due to its utility in determining the presence of specific themes and words within a qualitative data set, while minimizing the opportunity for bias from reviewers (Bengtsson, 2016). To facilitate this analysis, a hierarchal coding frame was created by manually reviewing the responses and establishing categories and tags through inductive coding (Bengtsson, 2016). The first level of classification identified the stakeholder group most pertinent in the response. Tags were then devised to sub-classify the issues identified in the responses into themes and sub-themes under each stakeholder. These classifications were designed in an attempt to be exhaustive and were not mutually exclusive between each stakeholder group, as different groups could face the same ethical issue within a different context.

Once responses went through an initial review and translation (where needed), reviewers (PG, TN, DB) looked at responses independently to avoid bias and influencing one another (O’Connor & Joffe, 2020). After the responses were reviewed individually, the reviewers used a consensus protocol to identify which stakeholder group (resident, family, staff, or organization) was primarily affected by the identified issue, as well the coding tags to be used to categorize the specific issue referenced into themes and sub-themes. This information and hierarchal coding frame was then reviewed and finalized by two Project Leads (PC and JO). Reviewers then independently coded individual responses using the agreed upon themes and sub-themes. The intercoder reliability was measured based on percent agreement prior to reviewing disagreements (O’Connor & Joffe, 2020). Intercoder reliability was measured for each of the four stakeholders’ who were discussed within responses. For responses where organizations were the stakeholder, there was 96% agreement among coders. Responses pertaining to staff had a 90% agreement between coders, while family and residents had an 89% and 80% agreement respectively. Overall, there was an 88.8% agreement among coders for all survey responses. The group then reviewed the results to ensure reliability of results and settled disagreements on the assigned code through consensus (O’Connor & Joffe, 2020).
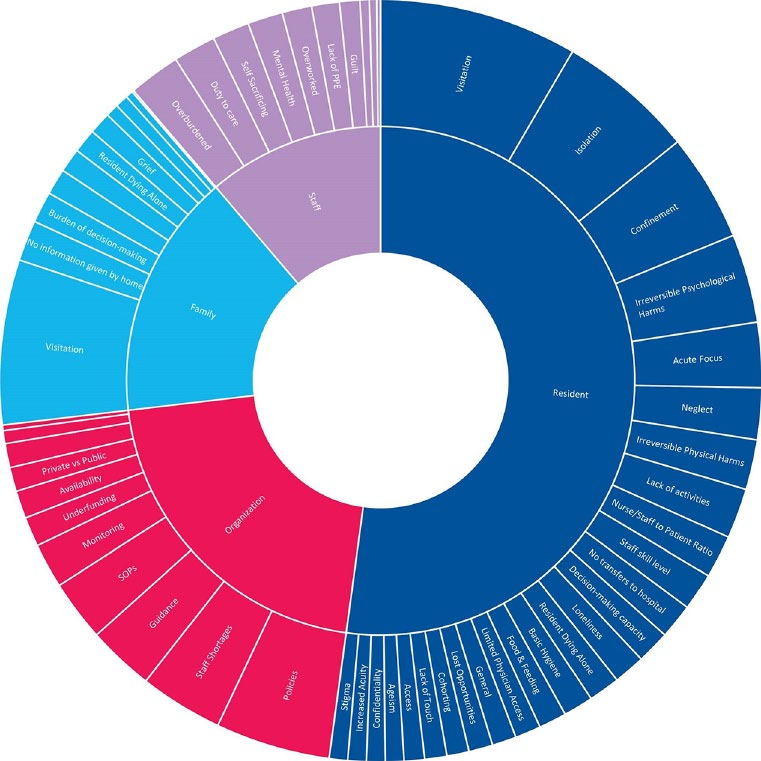


The tags assigned to each response were then translated into binary coding (0 or 1) to help facilitate a quantitative analysis of the issues mentioned in the responses. The quantitative analysis evaluated the frequency of the themes and sub-themes identified in the data set, and compared results across country type (HIC vs. LMIC) and stakeholder group in an effort to see commonalities and differences in the ethical issues faced in long-term care homes around the world.

## Results

The 284 responses included in the analysis were from 41 different countries. Using multiple World Bank resources, it was determined that 232 of these responses referred to LTC homes in a HIC, while 52 referred to LTC homes in LMICs. The focus of this analysis was to determine what stakeholders identified as ethical issues in LTC homes across the globe. Due to the bulk of responses obtained coming from HIC stakeholders, the major themes identified are broken into two categories: geographical location, and the stakeholder to which each ethical issue was most pertinent (Table 1).

**Table 1.** Survey Responses by Country Type and Stakeholder Group.

Country type	Number of responses	%	Survey responses identifying at least one. . .			
			Resident issue	Family issue	Staff issue	Organizational issue
HIC	232	81.7	204 (87.9%)	105 (45.3%)	61 (26.3%)	105 (45.3%)
LMIC	52	18.3	44 (84.6%)	15 (28.8%)	23 (44.2%)	29 (55.8%)
Total	284	100.0	248 (87.3%)	120 (42.3%)	84 (29.6%)	134 (47.2%)

### High-Income Countries

#### Residents

A wide array of ethical issues were identified from HIC respondents (Table 2). The most common ethical issues in survey responses included visitation (41%; *N* *=* 96), isolation of residents (27%; *N* *=* 63), confinement to rooms (22%; *N* *=* 51), psychological harm (20%; *N* *=* 20%), physical harm due to changes in care (12%; *N* *=* 27), and lack of activities (10%; *N* *=* 24).

**Table 2.** Ethical Issues Identified Primarily Affecting Residents.

Issues primarily affecting residents	HIC responses		LMIC responses		Total responses	
	*n*	%	*n*	%	*n*	%
Visitation	96	38.7	11	21.2	107	37.7
Isolation	63	25.4	11	21.2	74	26.1
Confinement	51	20.6	7	13.5	58	20.4
Irreversible psychological harms	47	19.0	1	1.9	48	16.9
Acute focus	23	9.3	12	23.1	35	12.3
Neglect	20	8.1	8	15.4	28	9.9
Lack of activities	24	9.7	3	5.8	27	9.5
Irreversible physical harms	27	10.9	0	0.0	27	9.5
Nurse/staff to patient ratio	19	7.7	4	7.7	23	8.1
Staff skill level	17	6.9	3	5.8	20	7.0
No transfers to hospital	15	6.0	4	7.7	19	6.7
Decision-making capacity	17	6.9	1	1.9	18	6.3
Resident dying alone	16	6.5	1	1.9	17	6.0
Loneliness	13	5.2	4	7.7	17	6.0
Basic hygiene	14	5.6	2	3.8	16	5.6
Food and feeding	14	5.6	1	1.9	15	5.3
Limited physician access	8	3.2	6	11.5	14	4.9
General	12	4.8	1	1.9	13	4.6
Lost opportunities	12	4.8	1	1.9	13	4.6
Cohorting	12	4.8	0	0.0	12	4.2
Lack of touch	11	4.4	1	1.9	12	4.2
Forced treatment	8	3.2	3	5.8	11	3.9
Access	7	2.8	4	7.7	11	3.9
Increased acuity	10	4.0	0	0.0	10	3.5
Ageism	7	2.8	3	5.8	10	3.5
Confidentiality	3	1.2	7	13.5	10	3.5
Stigma	4	1.6	6	11.5	10	3.5
Prioritization	6	2.4	2	3.8	8	2.8
Advanced care planning (ACP)	7	2.8	0	0.0	7	2.5
Restraints	6	2.4	1	1.9	7	2.5
Resident non-compliant	6	2.4	0	0.0	6	2.1
Falls	6	2.4	0	0.0	6	2.1
Medical focus of care	4	1.6	2	3.8	6	2.1
Physician skill level	6	2.4	0	0.0	6	2.1
Increased costs—general	4	1.6	1	1.9	5	1.8
Abandonment	4	1.6	0	0.0	4	1.4
Other	2	0.8	2	3.8	4	1.4
Language barriers	3	1.2	0	0.0	3	1.1
Safety	0	0.0	2	3.8	2	0.7
Discharges prevented	0	0.0	2	3.8	2	0.7
Access to spiritual care	1	0.4	0	0.0	1	0.4
Code Blue (PPE)	1	0.4	0	0.0	1	0.4

#### Family

Within HICs survey responses, the most frequently cited ethical issue pertaining to family members (Table 3) was visitation (34%; *N* *=* 80). Though not referenced as often, the other ethical issues included families not being given information by the home (9%; *N* *=* 22), the burden of decision-making (7%; *N* *=* 16), inconsistency of rule enforcement in LTCs (5%; *N* *=* 12), grief (5%; *N* *=* 12), lack of communication with loved ones (5%; *N* *=* 12), and fears over residents dying alone (5%; *N* *=* 12).

**Table 3.** Ethical Issues Identified Primarily Affecting Family.

Issues primarily affecting family	HIC responses		LMIC responses		Total responses	
	*n*	%	*n*	%	*n*	%
Visitation	80	32.3	9	17.3	89	31.3
No information given by home	22	8.9	0	0.0	22	7.7
Burden of decision-making	16	6.5	1	1.9	17	6.0
No communication with resident	12	4.8	2	3.8	14	4.9
Perceived inconsistency in rules or application (family vs. staff)	12	4.8	1	1.9	13	4.6
Resident dying alone	12	4.8	1	1.9	13	4.6
Grief	12	4.8	0	0.0	12	4.2
Irreversible psychological harms	7	2.8	0	0.0	7	2.5
Lost opportunities	6	2.4	1	1.9	7	2.5
Perceived inconsistency in rules or application (home vs. government)	5	2.0	0	0.0	5	1.8
Fear of LTC	5	2.0	0	0.0	5	1.8
Increased costs—general	4	1.6	1	1.9	5	1.8
Access to PPE—general	2	0.8	2	3.8	4	1.4
Access	2	0.8	2	3.8	4	1.4
Prioritization	1	0.4	0	0.0	1	0.4
Guilt	1	0.4	0	0.0	1	0.4

#### Staff

Ethical issues pertaining to staff were similarly distributed during the pandemic (Table 4). The most often cited issues included being overburdened (10%; *N* *=* 23), mental health challenges (7%; *N* *=* 16), being overworked (6%; *N* *=* 15), sacrificing health for work (6%; *N* *=* 14), and their duty to care as a staff (6%; *N* *=* 14).

**Table 4.** Ethical Issues Identified Primarily Affecting Staff.

Issues primarily affecting staff	HIC responses		LMIC responses		Total responses	
	*n*	%	*n*	%	*n*	%
Overburdened	23	9.3	5	9.6	28	9.9
Duty to care	14	5.6	9	17.3	23	8.1
Self sacrificing	14	5.6	6	11.5	20	7.0
Mental health	16	6.5	3	5.8	19	6.7
Overworked	15	6.0	1	1.9	16	5.6
Lack of PPE	9	3.6	6	11.5	15	5.3
Guilt	8	3.2	3	5.8	11	3.9
Pay (low/expectations)	7	2.8	2	3.8	9	3.2
Inability to follow IPAC	3	1.2	5	9.6	8	2.8
Lack of information	6	2.4	2	3.8	8	2.8
Work-life balance	5	2.0	1	1.9	6	2.1
Grief	5	2.0	0	0.0	5	1.8
Burnout	5	2.0	0	0.0	5	1.8
Seniority	4	1.6	0	0.0	4	1.4
Access	3	1.2	1	1.9	4	1.4
No sick days	3	1.2	1	1.9	4	1.4
Prioritization	4	1.6	0	0.0	4	1.4
Forced treatment	3	1.2	0	0.0	3	1.1
Working sick	3	1.2	0	0.0	3	1.1
Information sharing (family/resident)	2	0.8	1	1.9	3	1.1
Irreversible psychological harms	2	0.8	0	0.0	2	0.7
No respite	1	0.4	0	0.0	1	0.4
Safety	1	0.4	0	0.0	1	0.4

#### Organizations

Many ethical issues were described for organizations managing LTCs during the pandemic (Table 5). These issues primarily pertained to policymaking and enforcement (19%; *N* *=* 44), staffing shortages (18%; *N* *=* 42), guidance (11%; *N* *=* 26), standard operating practices (SOPs) (9%; *N* *=* 21), and monitoring the pandemic and resident health (7%; *N* *=* 16).

**Table 5.** Ethical Issues Identified Primarily Affecting Organizations.

Issues primarily affecting organizations	HIC responses		LMIC responses		Total responses	
	*n*	%	*n*	%	*n*	%
Policies	44	17.7	18	34.6	62	21.8
Staff shortages	42	16.9	3	5.8	45	15.8
Guidance	26	10.5	10	19.2	36	12.7
SOPs	21	8.5	12	23.1	33	11.6
Monitoring	16	6.5	8	15.4	24	8.5
Underfunding	8	3.2	8	15.4	16	5.6
Availability	12	4.8	3	5.8	15	5.3
Skill level of administrators—general	12	4.8	1	1.9	13	4.6
Private vs. public	10	4.0	3	5.8	13	4.6
Financial impact—general	5	2.0	2	3.8	7	2.5
Recruitment	6	2.4	0	0.0	6	2.1
Reputational risk	5	2.0	0	0.0	5	1.8
Distribution	3	1.2	2	3.8	5	1.8
Allocation	3	1.2	1	1.9	4	1.4
Access	2	0.8	1	1.9	3	1.1
Delays in funding	2	0.8	0	0.0	2	0.7

### Low and Middle-Income Countries

The survey results from LMICs present preliminary data on the ethical issues faced in global south LTCs during the pandemic. These responses are categorized based on the stakeholder most involved in the survey response.

#### Residents

The top ethical issues referencing residents in LMIC LTCs were an acute focus on care (23%; *N* *=* 12), visitation (21%; *N* *=* 11), and isolation (21%; *N* *=* 11) (Table 2).

#### Family

The ethical issues referencing families in LMIC LTCs were visitation (17%; *N* *=* 9), access to facility (4%; *N* *=* 2), and access to PPE (4%; *N* *=* 2) (Table 3).

#### Staff

The ethical issues referencing staff in LMIC LTCs were numerous, including duty to care (17%; *N* *=* 9), sacrificing health for work (12%; *N* *=* 6), and access to PPE (12%; *N* *=* 6) (Table 4).

#### Organization

The top ethical issues referencing organizations in LMIC LTCs included policymaking and enforcement (35%; *N* *=* 18), standard operating procedures (23%; *N* *=* 12), and guidance (19%; *N* *=* 10) (Table 5).

## Discussion

The purpose of this study was to present the ethical issues identified by LTC stakeholders across the globe during the COVID-19 pandemic The results reveal that most frequently identified ethical issues affecting residents including visitation, followed by isolation and then policies (Table 2). As an ethical issue, visitation was unique in that it appeared within the top three issues for both HIC and LMIC responses, individually and combined. In addition to visitation, the ethical issues of isolation and confinement were among the top three ethical issues primarily affecting residents for both HIC and LMIC responses individually and combined. Ethical issues that primarily affect family were not as frequently mentioned in LMIC responses; however, the top ethical issue reported for LMICs and HICs, both individually and combined, was visitation (Table 3). The greatest divergence between LMIC and HIC responses was in relation to ethical issues primarily affecting staff (Table 4). For HICs, top ethical issues primarily affecting staff were overburdened workforces, mental health, and overworked providers. For LMIC responses, ethical issues primarily affecting staff were duty to care, self-sacrificing and lack of PPE. Top ethical issues primarily affecting organizations were policy making and enforcement for both LMIC and HIC combined, followed by staff shortages and guidance (Table 5). In general, this report suggests that there is sufficient overlap between the identification of ethical issues faced by stakeholders during the COVID-19 pandemic and the existing literature, to potentially inform global ethical guidance.

### Visitation

Visitation has been a constant challenge during the COVID-19 pandemic, with many LTCs struggling to improve families’ access to loved ones despite the physical, psychological, and emotional benefits of visitation for residents (Freidus et al., 2020). Thus, it is unsurprising that is one of the most often mentioned issues for several stakeholders. While visitation was explicitly mentioned throughout survey responses, a number of other issues mentioned are directly connected to visitation, including a lack of communication from homes and fears of the psychological and physical harms associated with a lack of emotional connection with loved ones (Faghanipour et al., 2020). While families and residents receive the most consideration when it comes to the challenges of decreased, or non-existent, visitation policies, LTC staff faced challenges themselves. For residents who do not have family present, staff members often represent their closest bond on a day-to-day basis as both caregivers and their emotional support (Szczerbińska, 2020). As a result of visitation policies that did not prioritize family involvement until residents were often entering palliative care settings in LTC, the literature shows that physically and emotionally overburdened healthcare staff faced psychological challenges due to the rate and nature of deaths in LTC (Giesbrecht et al., 2021; Raza et al., 2020; Szczerbińska, 2020).

This correlates with the challenges faced at an organizational level, as the literature reflects a failure on the part of LTC governance to account for the importance of visitation while focussing more on infection prevention and control (IPAC) practices that ultimately led to a great deal of psychological and physical harm and distress for residents, families, and staff (Estabrooks et al., 2020; Faghanipour et al., 2020). It must also be noted that families stated within survey responses that the burden of important decision-making was often difficult during the pandemic, likely exacerbated by their lack of physical contact with residents who their decisions would impact.

### Isolation

Physical isolation of older adults is associated with increased rates of morbidity and mortality, with markedly reduced health outcomes specifically in LTC settings (Friedler et al., 2015; Parekh de Campos & Daniels, 2021). Due to the shared living environment in many LTCs, efforts to mitigate the spread of coronavirus among vulnerable populations during the height of the pandemic resulted in progressively restrictive IPAC practices in many LTCs, posing medico-legal ethical dilemmas (Liddell et al., 2021; Parekh de Campos & Daniels, 2021). Residents faced forced confinement across global LTCs, with a notable number of survey responses stating that confinement of loved ones was a major ethical challenge during the pandemic. For individuals with dementia, this has been shown to be particularly damaging to health and well-being (Iaboni et al., 2020). In restricting the physical movements of LTC residents, the ability for residents to self-determine their navigation of their space was notably absent, due to the risks posed to the welfare of their community members around them. While the ethical principles of social accountability and self-determination are often debated in the context of COVID-19 policies in LTC, there is a growing body of evidence showing that the effects of isolation during the pandemic has caused irreversible psychological and physical harms not only to residents, but to their family and staff caregivers (Parekh de Campos & Daniels, 2021).

### Staff and Organizational Issues

Globally, it is apparent that LTCs were unequipped to manage the pandemic due to issues with key areas of health care delivery. While organizations put in place many restrictive policies to reduce the spread of COVID-19, many of these efforts were complicated by gaps in resource allocation, many of which have been evident in the LTC sector for decades (Estabrooks et al., 2020). The reduced health and human resources available in LTCs, both in HICs and LMICs, is a longstanding issue that is part of a myriad of factors that precipitated the crisis in LTC homes during the pandemic (Estabrooks et al., 2020). Poor financing systems and a lack of political will to improve the infrastructure of LTC homes has culminated in a fractured system where IPAC policies were prioritized over longstanding issues in the quantity of health and human resources available, PPE access, and living and working conditions for staff who commit their time to work in LTCs (Estabrooks et al., 2020; Webster, 2021). As a result of the current state of the LTC system it is unsurprising that in LMICs, where infrastructure for LTCs is not as well funded as in HICs, that the top ethical issues facing staff involved sacrificing mental and physical health in order to continue providing care despite a lack of PPE, staff, and resources for care.

## Limitations

There are several limitations in the survey including that we could not validate the respondents as LTC stakeholders. Most respondents were from the high income countries despite efforts to seek input from stakeholders in low-middle income countries. Potential recruitment relied on a snowball strategy that could have been limited by the 41 day time period. This may not have allowed for a greater number of responses from LMICs. In addition to this, potential respondents might not have been able to participate in the survey due to language barriers, access to technology, or internet access needed to complete the survey. Furthermore, because our team is situated in Canada, we were able to reach survey participants from within Canada by drawing on our existing networks and relationships. Reaching stakeholders outside of Canada proved more challenges because we had few connections and less ability directly contact potential LTC stakeholder groups/survey participants. Lastly, in the statistical representation of HICs and LMICs, we did not categorize the differences within HICs and LMICs as it pertains to sociocultural elements.

## Conclusion

The COVID-19 pandemic challenged an already overburdened LTC system in need of transformation. In general, the survey showed there was a wide range of ethical issues identified by those in LTCs that must be addressed at both local and global levels. As part of any robust response to a pandemic and beyond, it is important to understand the ethical issues from the point of view of those who face them. We were curious about what were identified as ethical issues for stakeholders in the LTC homes they were associated with. Though there are limitations to some of the data collected in this survey, the findings point to further opportunities to validate the themes revealed in the survey, particularly with regard to visitation, isolation, duty to care, and issues related to staffing including duty to care, self-sacrificing and lack of PPE. While some of the issues identified were present prior to the pandemic, many were exacerbated in a manner that organizations, staff, families and residents could not adequately address. Though many of the policies put in place during the pandemic were developed in order to reduce pain and suffering, there is further opportunity to explore how restrictions may have compromised basic needs of residents, families, and staff, resulting in worsened health outcomes for LTC residents and a weakened health system.
